# Prevalence of polypharmacy and medication-related quality of life among adult patients in Al-Ahsa, Saudi Arabia

**DOI:** 10.25122/jml-2023-0101

**Published:** 2023-09

**Authors:** Abdullah Abdulaziz Alnaim, Sukainah Musa Almuhanna, Arwa Khalid AlHussain, Nurah Abdullatef Alkhteeb, Zainab Abdrabulridha Alabdullah

**Affiliations:** 1 Department of Family and Community Medicine, King Faisal University, Al-Ahsa, Kingdom of Saudi Arabia; 2 College of Medicine, King Faisal University, Al-Ahsa, Kingdom of Saudi Arabia

**Keywords:** polypharmacy, medication, quality of life, prevalence

## Abstract

Polypharmacy, often defined as the concurrent use of five or more medications, has become increasingly common due to various factors, including shifts in lifestyle and a rise in health-related issues among individuals. However, using multiple medications could bring more issues to the patient, as it is linked to poor health outcomes, including medication nonadherence, adverse pharmacological effects, and decreased quality of life (QoL). This study aimed to determine the prevalence of polypharmacy and identify drug-related problems among adult patients in Al-Ahsa. A cross-sectional study was conducted among adult patients living in Al-Ahsa, Saudi Arabia, taking five or more medications. A self-administered questionnaire was distributed among the target population using an online survey. The questionnaire included sociodemographic data (i.e., age, sex, education, etc.), a questionnaire to assess behaviors regarding the use of polypharmacy, and a 10-item questionnaire to measure medication-related quality of life (MRQoL). In total, 196 of the 1,088 patients surveyed took five or more medications, indicating an 18% prevalence of polypharmacy. Among the 196 patients, 26.5% reported poor medication-related QoL. In univariate analysis, sex, occupational status, average monthly income, hypertension, asthma, difficulty managing medications, and side effects experienced were significantly associated with MRQoL. Independent significant predictors of poor MRQoL were having asthma and difficulty managing medications. The prevalence of poor medication-related quality of life among adult patients in our region was 26.5%, lower than that in previous studies. Poor MRQoL was associated with lower monthly income, hypertension, asthma, side effects, and difficulty managing medications.

## INTRODUCTION

Polypharmacy is the concurrent use of several medications. There is no consensus regarding the definition, as it has been variably defined as either minor (two drugs) or major (five drugs). However, the most prevalent definition of polypharmacy is the simultaneous use of five or more medications [1-3]. Due to changes in lifestyle, general health, and the development of medical care, adults face health issues requiring more medications to treat. Polypharmacy among adults has become more common [4-6]. This issue arises because of their susceptibility to having more than one medical condition that necessitates pharmacotherapy [7-11].

Using numerous medications could bring more issues to the patient using them. For example, drug interactions can cause side effects that negatively affect the patient physically [12, 13]. Polypharmacy is linked to poor health outcomes, such as medication nonadherence, adverse pharmacological effects, and decreased quality of life (QoL) [14, 15]. The guidelines for hospitalized individuals with multimorbidity and long-term medication use require frequent reviews to improve QoL. In 2015, a medication-related quality-of-life measure was created to evaluate the quality of life of patients who take multiple medications. Although this problem is not new, medication-related outcomes affecting quality of life (MRQoL) are poorly addressed [14, 16].

There are studies regarding health-related quality of life and quality of life in general. However, a few studies address medication-related quality of life in Europe and Ethiopia. Unfortunately, there are few studies on this issue in Saudi Arabia [17-20]. When two medications known to interact are prescribed simultaneously, regardless of whether adverse reactions occur, this is considered a drug interaction [21].

Older people are more likely to experience drug interactions [22]. This higher susceptibility is attributed to physiologic changes associated with ageing that impact the pharmacokinetic and pharmacodynamic characteristics of various medicines. These modifications may arise from genetics, ingrained lifestyle choices, and/or the environment, which could increase interpatient variability and complicate drug interaction management in the elderly [21].

The extent of potential drug interactions in the older population is not well-documented [21]. However, increased drug use is associated with a higher risk of potential drug interactions, resulting in many visits to the emergency room and medical offices. Research indicates that drug interactions can reduce the functional capacity of older individuals and contribute to increased morbidity, often undiagnosed in clinical practice [23].

Therefore, this study aimed to determine the prevalence of polypharmacy and to identify medication-related quality of life problems among adult patients in the Eastern Province.

## MATERIAL AND METHODS

### Study design and location

This cross-sectional study aimed to assess medication-related quality of life among residents of Al-Ahsa, Saudi Arabia, aged 18 years or older who were using five or more medications.

### Sample size

The sample size was calculated using the formula *n=z2pq\d2*, with a confidence level of 95%, an estimated proportion of 50%, and a 5% level of precision, resulting in an appropriate sample size of 384.

### Inclusion and exclusion criteria

The study included all residents of Al-Ahsa, Saudi Arabia, who met the following criteria: aged 18 years or older, using five or more medications, and able to complete the questionnaire. Individuals who were not residing in Al-Ahsa, used fewer than five medications, did not complete the questionnaire, or were less than 18 years old were excluded from the study.

### Data collection and management

An online survey was adapted from previous studies [19, 20, 24] (**Supplementary material**) and disseminated to Al-Ahsa residents through various social media applications. The data were stored in IBM SPSS version 23 and some cloud storage applications such as Google Drive. No private personal information, such as name or ID number, was recorded to maintain the participants' privacy and confidentiality.

Supplementary material

### Statistical analysis

Medication-related quality of life assessment followed the method used in the study by Tegegn *et al*. [19], with cutoff points at the 33rd and 66th percentiles to determine the severity of impairment. The overall MRQoL cutoff point was the 50th percentile. Data are presented as numbers and percentages (%). Inferential statistics (univariate and multivariate analyses) were conducted to determine the influence of poor MRQoL with corresponding odds ratios and 95% confidence intervals. A p-value of less than 0.05 (two-sided) was used to indicate statistical significance. All statistical analyses were performed using the Statistical Packages for Software Sciences (SPSS) version 26 (Armonk, New York, IBM Corporation).

## RESULTS

We received 1,088 completed surveys. A total of 892 of the respondents were taking fewer than five medications and were excluded for not meeting the inclusion criteria. The remaining 196 cases met the inclusion criteria for polypharmacy (taking 5 or more medications), giving an overall prevalence of polypharmacy of 18%. Table 1 presents the sociodemographic characteristics of the 196 adult patients. A total of 26.5% were older than 60, with more than half being females (52.6%) and 59.7% having a university degree. Nearly 40% of respondents were retired employees, and 56.6% earned more than 10,000 SAR monthly. The majority of the patients were married (79.6%). In addition, 41.8% of the patients were obese.

**Table 1:** Sociodemographic characteristics of patients with polypharmacy (n=196)

Study variables	N (%)
**Age group**	
19-30 years	21 (10.7%)
31-40 years	19 (09.7%)
41-50 years	43 (21.9%)
51-60 years	61 (31.1%)
>60 years	52 (26.5%)
**Gender**	
Male	93 (47.4%)
Female	103 (52.6%)
**Education level**	
Primary	25 (12.8%)
Intermediate	17 (08.7%)
Secondary	37 (18.9%)
University or higher	117 (59.7%)
**Occupational status**	
Employed	60 (30.6%)
Unemployed	44 (22.4%)
Housewife	04 (02.0%)
Retired	76 (38.8%)
Student	12 (06.1%)
**Average monthly income (SAR)**	
<2,500	22 (11.2%)
2,500–4,999	26 (13.3%)
5,000–10,000	37 (18.9%)
>10,000	111 (56.6%)
**Marital status**	
Single	30 (15.3%)
Married	156 (79.6%)
Separated	10 (05.1%)
**BMI level**	
Underweight (<18.5 kg/m^2^)	06 (03.1%)
Normal (18.5–24.9 kg/m^2^)	36 (18.4%)
Overweight (25–29.9 kg/m^2^)	72 (36.7%)
Obese (≥30 kg/m^2^)	82 (41.8%)

**Figure 1:**
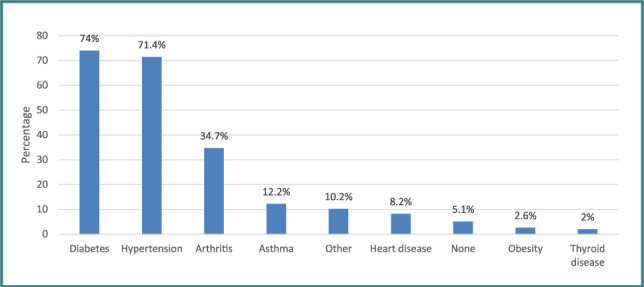
Prevalence of chronic diseases among patients'

The most commonly associated chronic diseases were diabetes (74%), followed by hypertension (71.4%) and arthritis (34.7%), while the least common was thyroid disease (2%) (Figure 1).

As shown in Table 2, more than half of the patients (54.6%) were taking antidiabetic and antihypertensive medications. Nearly all (90.3%) indicated they always took medicines on time. Only 14.8% expressed having difficulty managing medications. However, 33.7% experienced side effects after taking medications, and the most common were dizziness/nausea/constipation (15.2%).

**Table 2:** Patients’ behaviors regarding the use of polypharmacy medication (n=196)

Variables	N (%)
**Type of medication being used**	
Antidiabetic	36 (18.4%)
Anti-HTN	39 (19.9%)
Anti-DM and anti-HTN	107 (54.6%)
Vitamins and supplements	06 (03.1%)
Others	08 (04.1%)
**Do you take the medications regularly?**	
Yes, always on time	177 (90.3%)
Sometimes missed	02 (01.0%)
No	17 (08.7%)
**Is there any difficulty managing all these medications?**	
Yes	29 (14.8%)
Occasionally	86 (43.9%)
No	81 (41.3%)
**Did you develop any side effects from these medications**	
Yes	66 (33.7%)
No	130 (66.3%)
**If yes, what are the side effects did you experience? (n=66)**	
Dizziness/Nausea/Constipation	10 (15.2%)
Fatigue	06 (09.1%)
Headache	06 (09.1%)
Joint and muscle pain	08 (12.1%)
Abdominal pain	05 (07.6%)
Others	31 (47.0%)

In Table 3, severe impairment of role limitation, self-control, and overall QoL was found in 17.3%, 10.2%, and 12.8%, respectively. The prevalence of patients with poor medication-QoL was 26.5%.

**Table 3:** Classification of impairment using MRQoL (n=196)

Severity of impairment	N (%)
**Role limitation due to medication**	
None/Mild impairment (score 0–3)	84 (42.9%)
Moderate impairment (score 4–6)	78 (39.8%)
Severe impairment (score 7–10)	34 (17.3%)
**Self-control**	
None/Mild impairment (score 0–3)	95 (48.5%)
Moderate impairment (score 4–6)	81 (41.3%)
Severe impairment (score 7–10)	20 (10.2%)
**Overall MRQoL score**	
None/Mild impairment (score 0–3)	84 (42.9%)
Moderate impairment (score 4–6)	87 (44.4%)
Severe impairment (score 7–10)	25 (12.8%)
**Level of MRQoL**	
Poor (score 11–20)	52 (26.5%)
Good (score 0–10)	144 (73.5%)

Measuring the relationships among the level of MRQoL, sociodemographic characteristics and the patient's behavior toward polypharmacy (Table 4) revealed that the prevalence of patients with poor MRQoL was significantly higher among females (p=0.031), the unemployed (p=0.041), those with a lower monthly income (p=0.001), hypertension (p=0.036), asthma (p=0.001), difficulty or occasional difficulty in managing medications (p<0.001), and those who experienced side effects from the medications taken (p=0.010).

**Table 4:** Relationship between the level of MRQoL according to sociodemographic characteristics and behavior toward polypharmacy (n=196)

Factor	Level of MRQoL		p value^§^
	Poor N (%) (n=52)	Good N (%) (n=144)	
**Age group**			
≤50 years	24 (46.2%)	59 (41.0%)	0.517
>50 years	28 (53.8%)	85 (59.0%)	
**Gender**			
Male	18 (34.6%)	75 (52.1%)	0.031 **
Female	34 (65.4%)	69 (47.9%)	
**Educational level**			
Secondary or below	23 (44.2%)	56 (38.9%)	0.501
University or higher	29 (55.8%)	88 (61.1%)	
**Occupational status**			
Employed/Student	13 (25.0%)	59 (41.0%)	0.041**
Unemployed	39 (75.0%)	85 (59.0%)	
**Average monthly income (SAR)**			
≤10,000	33 (63.5%)	52 (36.1%)	0.001**
>10,000	19 (36.5%)	92 (63.9%)	
**Marital status**			
Unmarried	14 (26.9%)	26 (18.1%)	0.174
Married	38 (73.1%)	118 (81.9%)	
**BMI level**			
Normal or underweight (<25 kg/m^2^)	15 (28.8%)	27 (18.8%)	0.221
Overweight (25–29.9 kg/m^2^)	15 (28.8%)	57 (39.6%)	
Obese (≥30 kg/m^2^)	22 (42.3%)	60 (41.7%)	
**Associated chronic disease ^†^**			
Hypertension	43 (82.7%)	97 (67.4%)	0.036**
Diabetes	38 (73.1%)	107 (74.3%)	0.863
Asthma	13 (25.0%)	11 (07.6%)	0.001**
Arthritis	19 (36.5%)	49 (34.0%)	0.744
Heart disease	07 (13.5%)	09 (06.3%)	0.104
**Having difficulty managing medications**			
Yes/Occasionally	46 (88.5%)	69 (47.9%)	<0.001**
No	06 (11.5%)	75 (82.1%)	
**Experienced side effects from taking medications**			
Yes	25 (48.1%)	41 (28.5%)	0.010**
No	27 (51.9%)	103 (71.5%)	

† Some patients have multiple chronic diseases.

§ P value was calculated using the chi-square test.

** Significant at the p<0.05 level.

A multivariate regression model was subsequently conducted to determine the significant independent predictor of poor MRQoL (Table 5). The results found that having asthma and having difficulty or occasional difficulty managing medications were significant independent predictors of poor MRQoL while having a higher monthly income was a significant independent predictor of good MRQoL. This further suggests that compared to patients without asthma, patients with asthma were predicted to have an increased risk of poor MRQoL by at least 3.7 times (AOR=3.714; 95% CI=1.395–9.888; p=0.009). Patients who had difficulty managing medications were 6 times more likely to have poor MRQoL than patients who did not have difficulty (AOR=6.009; 95% CI=2.264–15.951; p<0.001). In contrast, compared to lower earners, patients with better monthly income were predicted to have a decreased risk of poor MRQoL by at least 55% (AOR=0.447; 95% CI=0.212–0.940; p=0.034). However, gender, occupational status, hypertension, and experiencing side effects due to medications did not significantly affect the level of QoL after adjustment in the regression model (p>0.05).

**Table 5:** Multivariate logistic regression analysis of the influence of poor MRQoL according to sociodemographic characteristics and behavior toward polypharmacy (n=196)

Factor	AOR	95% CI	p value
**Gender**			
Male	Ref		
Female	1.692	0.792-3.614	0.175
**Occupational status**			
Employed/Student	Ref		
Unemployed	1.625	0.719-3.671	0.243
**Average monthly income (SAR)**			
≤10,000	Ref		
>10,000	0.447	0.212-0.940	0.034**
**Hypertension**			
No	Ref		
Yes	2.294	0.890-5.911	0.086
**Asthma**			
No	Ref		
Yes	3.714	1.395-9.888	0.009**
**Having difficulty managing medications**			
No	Ref		
Yes/Occasionally	6.009	2.264-15.951	<0.001**
**Experienced side effects from taking medications**			
No	Ref		
Yes	1.366	0.636-2.933	0.423

AOR – Adjusted odds ratio; CI – Confidence interval;

** Significant at the p<0.05 level.

## DISCUSSION

This study evaluates the prevalence of polypharmacy and medication-related quality of life among adult patients in the Al-Ahsa Region, Saudi Arabia. According to the systematic review conducted by Masnoon *et al*. [2], the most common definition of polypharmacy is taking five or more medications daily. This study adopted this definition. Using similar criteria, we found that the prevalence of polypharmacy in this study was 18% (196 out of 1,088). Contradicting these reports, a lower prevalence of polypharmacy was reported in Riyadh, with prevalence rates of 51.5% and 46%, respectively [5, 14]. These findings have also been replicated in 24 countries, and the prevalence of polypharmacy among HIV patients was 42.1%. However, the excessive use of multiple medications has been noted among patients with advanced illnesses. The study indicated that approximately one-third of the patients took 14 or more medications daily [20]. The author emphasized the importance of shared decision-making toward deprescribing medications among adult patients with advanced conditions.

According to our results, 26.5% were considered to have a poor medication-related quality of life. This result is lower than the prevalence documented by Tegegn *et al*. [19], wherein 75.3% of older patients were found to have poor MRQoL. Regarding MRQoL domains, polypharmacy seems to have no greater effect on role limitations due to medication and self-control domains, with both fields showing better outcomes. Accordingly, we discovered that only 17.1% were considered severely impaired in terms of role limitation, and only 10.2% were considered severely impaired in self-control. The overall MRQoL was severely impaired in only 12.8% of the patients, while 42.9% had normal or mild impairment. This scenario is likely better than the study of Colombijn *et al*. [17]. Based on the reports, after adjusting for confounders, more medications were associated with a lower physical component score (PCS), mental component score (MCS), and self-rated health in dialysis patients with multiple symptoms. In India [25], after adjusting for potential confounders, a study found a negative correlation between the number of medications and the PCS-adjusted score. At the same time, there was a difference of 3.72 points in the unadjusted MCS domain. The author concluded that the outcome indicated that polypharmacy might lead to impaired physical ability, possibly due to increased drug-to-drug interactions, adverse drug reactions, frailty, and disability, often correlated with polypharmacy [26].

In our univariate analysis, several demographic variables emerged as factors influencing MRQoL. For example, poor MRQoL was significantly prevalent among females, unemployed individuals, those earning less, those who experienced side effects, and those with difficulty managing medications. However, in our multivariate estimates, lower monthly income and difficulty managing medications remained statistically significant and were determined to be independent predictors of poor MRQoL. These findings are not consistent with the report of Tegegn *et al*. [19]. According to their multivariate regression model, there was a one-unit increase in the frequency of hospital visits, and using more than five medications increased the likelihood of potential severe impairment of MRQoL by at least 1.91 times. However, they found no significant relationship between poor MQoL and the sociodemographic or clinical data of the patients.

Moreover, according to the cohort study performed by Okoli *et al*. [16], patients with polypharmacy had significantly worse health outcomes independent of existing comorbidities, which was supported by the paper of Schenker *et al*. [24]. In our study, poor MRQoL was significantly prevalent in patients with hypertension and asthma, consistent with previous reports.

Age was a factor in health-related quality of life among polypharmacy patients, as reported by Jennings *et al*. [15]. Based on their accounts, an ambulatory older patient cohort attending a specialist hospital-based geriatric medicine outpatient clinic experienced baseline polypharmacy and multimorbidity and reported poor age-adjusted health-related quality of life (HRQoL). Consistent with these findings, another cohort study conducted among older adults in Australia [27] observed that when measuring HRQoL from 2013 to 2017, older adults with persistent polypharmacy had the lowest HRQoL scores. In our study, although older age groups more often experienced poor MRQoL, the results did not reach statistical significance (p=0.517), which is inconsistent with previous results. The literature indicates the importance of age when prescribing medications to older patients, as polypharmacy may affect the overall treatment process. Hence, careful concern should be given to managing polypharmacy among older adults, especially those with advanced illnesses.

## CONCLUSION

The prevalence of poor medication-related quality of life due to polypharmacy was 26.5%. Insufficient monthly income, hypertension, asthma, difficulties managing medication, and experiencing side effects were some of the most detrimental factors associated with MRQoL. In addition, the severity of the illness may lead to taking more medications and lower quality of life. Therefore, healthcare providers should seek deprescribing methods to optimize drug therapy and minimize the polypharmacy-related poor quality of life in this setting.

## References

[1] Gnjidic D, Hilmer SN, Blyth FM, Naganathan V (2012). Polypharmacy cutoff and outcomes: five or more medicines were used to identify community-dwelling older men at risk of different adverse outcomes. J Clin Epidemiol.

[2] Masnoon N, Shakib S, Kalisch-Ellett L, Caughey GE (2017). What is polypharmacy? A systematic review of definitions. BMC Geriatrics.

[3] Guthrie B, Makubate B, Hernandez-Santiago V, Dreischulte T (2015). The rising tide of polypharmacy and drug-drug interactions: population database analysis 1995-2010. BMC Med.

[4] Melzer D, Tavakoly B, Winder RE, Masoli JA (2015). Much more medicine for the oldest old: trends in UK electronic clinical records. Age Ageing.

[5] Aljawadi MH, Khoja AT, Alaboud NM, AlEnazi ME, Al-Shammari SA, Khoja TA, AlMuqbil MS, Alsheikh AM, Alwhaibi M (2022). Prevalence of Polypharmacy and Factors Associated with it Among Saudi Older Adults–Results from the Saudi National Survey for Elderly Health (SNSEH). Saudi Pharm J.

[6] Fitzgerald LS, Hanlon JT, Shelton PS (1997). Reliability of a modified medication appropriateness index in ambulatory older persons. Ann Pharmacother.

[7] Gurwitz JH (2004). Polypharmacy: a new paradigm for quality drug therapy in the elderly?. Arch Intern Med.

[8] Jörgensen T, Johansson S, Kennerfalk A, Wallander MA, Svärdsudd K (2001). Prescription drug use, diagnoses, and healthcare utilization among the elderly. Ann Pharmacother.

[9] Rigler SK, Perera S, Jachna C, Shireman TI, Eng M (2004). Comparison of the association between disease burden and inappropriate medication use across three cohorts of older adults. Am J Geriatr Pharmacother.

[10] Safran DG, Neuman P, Schoen C, Kitchman MS (2005). Prescription drug coverage and seniors: findings from a 2003 national survey. Health Aff (Millwood).

[11] Gorard DA (2006). Escalating polypharmacy. QJM.

[12] Field TS, Gurwitz JH, Avorn J, McCormick D (2001). Risk factors for adverse drug events among nursing home residents. Arch Intern Med.

[13] Pharmaceutical Society of Australia Ltd (2011). Guidelines for Pharmacists Providing Home Medicines Review (HMR) Service. http://6cpa.com.au/medicationmanagement-programs/home-medicines-review.

[14] Balkhi B, AlQahtani N, Alwhaibi M, Alshammari TM (2021). Prevalence and Factors Associated With Polypharmacy Use Among Adult Patients in Saudi Arabia. J Patient Saf.

[15] Jennings ELM, O'Mahony D, Gallagher PF (2022). Medication-related quality of life (MRQoL) in ambulatory older adults with multi-morbidity and polypharmacy. Eur Geriatr Med.

[16] Okoli C, de Los Rios P, Eremin A, Brough G (2020). Relationship Between Polypharmacy and Quality of Life Among People in 24 Countries Living With HIV. Prev Chronic Dis.

[17] Colombijn JMT, Bonenkamp AA, van Eck van der Sluijs A, Bijlsma JA (2021). Impact of Polypharmacy on Health-Related Quality of Life in Dialysis Patients. Am J Nephrol.

[18] Tseng HM, Lee CH, Chen YJ, Hsu HH (2016). Developing a measure of medication-related quality of life for people with polypharmacy. Qual Life Res.

[19] Tegegn HG, Erku DA, Sebsibe G, Gizaw B (2019). Medication-related quality of life among Ethiopian elderly patients with polypharmacy: A cross-sectional study in an Ethiopia university hospital. PLoS One.

[20] Khoja AT, Aljawadi MH, Al-Shammari SA, Mohamed AG (2018). The health of Saudi older adults; results from the Saudi National Survey for Elderly Health (SNSEH) 2006-2015. Saudi Pharm J.

[21] Hines LE, Murphy JE (2011). Potentially harmful drug-drug interactions in the elderly: a review. Am J Geriatr Pharmacother.

[22] Mallet L, Spinewine A, Huang A (2007). The challenge of managing drug interactions in elderly people. Lancet.

[23] Marengoni A, Pasina L, Concoreggi C, Martini G (2014). Understanding adverse drug reactions in older adults through drug-drug interactions. Eur J Intern Med.

[24] Schenker Y, Park SY, Jeong K, Pruskowski J (2019). Associations Between Polypharmacy, Symptom Burden, and Quality of Life in Patients with Advanced, Life-Limiting Illness. J Gen Intern Med.

[25] Paramba T, Zilate S (2022). Cross-Sectional Observational Study on Association of Polypharmacy With Health-Related Quality of Life in Patients With Hypertension. Cureus.

[26] Halli-Tierney AD, Scarbrough C, Carroll D (2019). Polypharmacy: Evaluating Risks and Deprescribing. Am Fam Physician.

[27] Aljeaidi MS, Haaksma ML, Tan ECK (2022). Polypharmacy and trajectories of health-related quality of life in older adults: an Australian cohort study. Qual Life Res.

