# Supplementary material for: Prevalence of polypharmacy and medication-related quality of life among adult patients in Al-Ahsa, Saudi Arabia
Source: J Med Life. 2023 Sep;16(9):1415–20. doi: 10.25122/jml-2023-0101 (PMC10719800; doi:10.25122/jml-2023-0101)
Supplement: Supplementary file 1 [file JMedLife-16-1415-s001.pdf]

## Appendix

### Questionnaire:

| Question | Response |
|---|---|
| **Biographical data**<br>This questionnaire is intended for adult patient who take more than 3 medications aged above 18 years | |
| 1. Gender | • Male<br>• Female |
| 2. Age | • --------- |
| 3. Level of education | • Primary<br>• Intermediate<br>• Secondary<br>• University and above |
| 4. Occupational status | • Employed<br>• Unemployed<br>• Housewife<br>• Retired<br>• (Other) |
| 5. Average monthly income "Saudi Riyal" | • > 10.000<br>• 9999-7500<br>• 7499-5000<br>• 4999-2500<br>• <2500<br>• Missing |
| 6. Marital status | • Married<br>• Polygamy<br>• Single<br>• Separated<br>• Divorced |
| **Health condition** | |
| 7. Do you have Chronic condition | • Hypertension<br>• Diabetes<br>• Asthma<br>• Arthritis<br>• Heart disease<br>• Other |
| 8. Height | • --------- |
| 9. Weight | • --------- |
| 10. How many medications do you take? | • Less than five<br>• Five and more |
| 11. Do you take the medications regularly? | • Yes "always on time"<br>• Sometimes missed<br>• No |
| 12. Types of medication that you are used | • Antidepressant<br>• Antidiabetic<br>• Heart medications<br>• Psychiatric medications<br>• Neurological medications<br>• Eye medication<br>• Pain killer<br>• Asthma medication<br>• Joint pain medication<br>• Multivitamins<br>• GI medication<br>• Chronic lung medication<br>• Antihyperlipidemia<br>• ENT medication<br>• Others ... |


Licensed under CC BY 4.0

## Appendix

| | |
|---|---|
| 13. Is there any difficulty managing all these medication | • Yes<br>• Sometimes<br>• Rarely<br>• No |
| 14. Did you develop any side effects from these medications | • Yes<br>• No |
| 15. If yes, write what side effects did you experience | • --------- |
| **Medication - Related Quality of life**<br>Role limitations due to medication | |
| 1. Cut down the amount of time you spent on work or daily activities | • Always<br>• Sometimes<br>• Never |
| 2. Accomplish the work less than you would like | • Always<br>• Sometimes<br>• Never |
| 3. Were limited in the work or other daily activities | • Always<br>• Sometimes<br>• Never |
| 4. Took extra effort or had difficulty performing the work or daily activities | • Always<br>• Sometimes<br>• Never |
| 5. Interfered with your social activities with family or friends | • Always<br>• Sometimes<br>• Never |
| 6. Interfered with your recreational activities, such as exercise or watching TV | • Always<br>• Sometimes<br>• Never |
| **Self-control** | |
| 7. Felt frustrated or downhearted | • Always<br>• Sometimes<br>• Never |
| 8. Thought of yourself as a burden to others | • Always<br>• Sometimes<br>• Never |
| 9. Worried about disappointing others | • Always<br>• Sometimes<br>• Never |
| 10. Didn't do work or other activities as a result of medication problems | • Always<br>• Sometimes<br>• Never |
| 11. Reduced the number of days feeling full of pep | • Always<br>• Sometimes<br>• Never |

All participants were asked to obtain consent before being involved in the study. The information was used only for scientific purposes.


Licensed under CC BY 4.0
